# Deep Learning-Driven Single-Lead ECG Classification: A Rapid Approach for Comprehensive Cardiac Diagnostics

**DOI:** 10.3390/diagnostics15030384

**Published:** 2025-02-06

**Authors:** Mohamed Ezz

**Affiliations:** Department of Computer Sciences, College of Computer and Information Sciences, Jouf University, Sakaka 72388, Saudi Arabia; maismail@ju.edu.sa

**Keywords:** cardiovascular diseases (CVDs), single-lead ECG, deep learning models, cardiac condition classification, VGG16, telemedicine applications

## Abstract

**Background/Objectives**: This study aims to address the critical need for accessible, early, and accurate cardiac di-agnostics, especially in resource-limited or remote settings. By shifting focus from traditional multi-lead ECG analysis to single-lead ECG data, this research explores the potential of advanced deep learning models for classifying cardiac conditions, including Nor-mal, Abnormal, Previous Myocardial Infarction (PMI), and Myocardial Infarction (MI). **Methods**: Five state-of-the-art deep learning architectures—Inception, DenseNet201, MobileNetV2, NASNetLarge, and VGG16—were systematically evaluated on individual ECG leads. Key performance metrics, such as model accuracy, inference time, and size, were analyzed to determine the optimal configurations for practical applications. **Results**: VGG16 emerged as the most accurate model, achieving an F1-score of 98.11% on lead V4 with a prediction time of 4.2 ms and a size of 528 MB, making it suitable for high-precision clinical settings. MobileNetV2, with a compact size of 13.4 MB, offered a balanced performance, achieving a 97.24% F1-score with a faster inference time of 3.2 ms, positioning it as an ideal candidate for real-time monitoring and telehealth applications. **Conclusions**: This study bridges a critical gap in cardiac diagnostics by demonstrating the feasibility of lightweight, scalable, single-lead ECG analysis using advanced deep learning models. The findings pave the way for deploying portable diagnostic tools across diverse settings, enhancing the accessibility and efficiency of cardiac care globally.

## 1. Introduction

The advent of machine learning (ML) in cardiology heralds a transformative era in diagnosing and understanding cardiac diseases. Recent studies have illuminated the path toward automating the interpretation of ECG signals, which are pivotal for the early detection of heart conditions. Despite substantial progress, a noticeable gap exists in the research landscape—the exploration of ML models tailored to the classification of single ECG leads has not been thoroughly investigated. This gap underscores the novelty of our study, marking it as potentially the first of its kind to delve into the realm of single-lead ECG analysis using advanced machine learning techniques.

Naser et al. [1] illustrate some of the challenges associated with traditional diagnostic approaches and indicate the requirement for incorporating advanced ML algorithms for early detection of CVD and precise prognosis, which will contribute to better patient care and a reduction in morbidity and mortality within the population. In addition, Restrepo Tique et al. [2] investigate the latest technological developments and trends in the fields of diagnosis, treatment, and monitoring of CVDs through AI, telemedicine, and other digital healthcare technologies. The purpose of the study is to bring to light that these innovations can support better patient management and attain better clinical outcomes. According to Bhagawati et al. [3], hybrid deep learning (HDL) can be used to compute the risk related to CVD from characteristics obtained from carotid plaques. Their study suggests that HDL is more effective than both unidirectional and bidirectional deep learning, as well as more effective than traditional machine learning methods in predicting the risk of CVD. In addition, Azmi et al. [4] deliver a comparative evaluation of different ML approaches focused on analyzing data related to CVDs, particularly in classification and prediction forms, to improve the precision of identifying the highest risk of CVD and help researchers and medical experts with early diagnosis and precautions aimed at reducing mortality from heart diseases.

The previous studies predominantly concentrate on multi-lead ECG data or full ECG recordings, leveraging a diverse array of ML approaches. For instance, the ensemble methods combining AdaBoost and logistic regression in [5], and the deep learning framework surpassing cardiologist performance in [6], all utilize comprehensive ECG data. While these methodologies demonstrate robust diagnostic capabilities, they are inherently dependent on multi-lead setups, which require specialized equipment and significant computational resources. This dependency limits their scalability, particularly in remote monitoring or wearable technology contexts, where portability and efficiency are critical.

To the best of our knowledge, this study is the first to systematically evaluate the diagnostic potential of single-lead ECG data using advanced deep learning models. Single-lead systems offer unique advantages in terms of portability, cost-efficiency, and scalability, making them a promising alternative for cardiac diagnostics in resource-limited environments. By introducing this novel approach, our work aims to bridge a critical gap in ECG-based diagnostics and establish a foundation for leveraging deep learning in single-lead analysis.

Our contribution in this study is to embrace the inherent diagnostic capability of each ECG lead. Therefore, it is our intention to develop a novel method that introduces a completely new depth to the practice of ECG-based diagnostics using single-lead analysis, which could be truly revolutionary. This research pivots on a critical inquiry: Would deep learning models applied to individual ECG leads provide reliable and time-efficient diagnoses compared to traditional multi-lead approaches?

To address this question, our investigation encompasses several focal points:

1. Model Performance by ECG Lead: We dissect the performance of various deep learning models when applied to single-lead ECG data, assessing which leads offer the most diagnostic value when observed in isolation.
2. Model Performance by ECG Lead/Class: The analysis is deepened by evaluating how these models perform on different cardiac conditions—Normal, Abnormal, PMI, and MI—thus unraveling the specificity and sensitivity of each model–lead combination.
3. Performance/Time Analysis: We strike a delicate balance between diagnostic accuracy (F1-score), computational efficiency (prediction time), and model size, recognizing the importance of real-time analysis in clinical settings and wearable devices.
4. In-Depth Analysis of Optimal Model Performance: We hone in on the optimal model–lead pairing, highlighted by a thorough evaluation of its Receiver Operating Characteristic (ROC) curve and confusion matrix, to affirm its readiness for clinical application.

This structured inquiry, embedded within our introduction, sets the stage for a methodical and innovative exploration of single-lead ECG classification. It not only endeavors to fill the existing research void, but also aspires to stimulate a paradigm shift toward a more accessible and tailored diagnostic process, potentially redefining the standards of cardiac care.

The organization of the paper is structured as follows: Section 2 reviews the related work, discussing the existing literature and identifying the research gap addressed by this study. Section 3 details the methodology, describing the overall approach, dataset, preprocessing techniques, deep learning model integration, and experimental setup. Section 4 presents the results, evaluating the performance of different models across individual ECG leads. Section 5 provides a comprehensive discussion, analyzing the findings, comparing model performance, and exploring practical implications. Finally, Section 6 concludes the paper, summarizing the key outcomes, highlighting the potential impact of the proposed approach, and suggesting directions for future research.

## 2. Related Work

Previous studies on ECG classification encompass a wide range of methodologies, which can be broadly categorized into the following:

1. Feature-Based Traditional Machine Learning Methods: These approaches utilize manually extracted features, such as heart rate variability or frequency-domain transformations, as inputs for algorithms like support vector machines, logistic regression, and ensemble methods [5,6,7,8,9,10].
2. Deep Learning Approaches: Advanced architectures, including convolutional neural networks (CNNs), recurrent neural networks (RNNs), and hybrid models, have emerged as dominant techniques, due to their ability to automate feature extraction and improve classification performance [11,12,13,14,15,16,17,18,19].
3. Multi-Modal and Specialized Techniques: Recent studies integrate additional data sources, such as clinical annotations or multi-lead ECG data, with machine learning models to enhance diagnostic accuracy and interpretability [20,21,22,23,24,25,26,27].

This categorization highlights the breadth of prior research, and provides context for understanding the methodologies evaluated in this study. Unlike most prior works, our approach focuses specifically on single-lead ECG data, systematically evaluating multiple deep learning models to address the challenges of resource-constrained environments.

The classification and analysis of ECG images utilizing ML techniques have witnessed significant advancements, as evidenced by various research endeavors to enhance diagnostic accuracy and efficiency. This section outlines a taxonomy of notable methods and their performance metrics within ECG recognition.

Wang et al. [7] presented a risk prediction of cardiovascular disease risks using the ML approach. By applying logistic regression (LR) and factor analysis, the CVD risk assessment has been used to define the indexes of risk factors, including age, obesity, and life habits. It presents a two-staged approach based on K-means clustering and random forest, with the potential to enhance the accuracy of CVD risk prediction compared to conventional approaches. Rastogi and Girerd [8] presented machine learning-based survival prediction models for cardiovascular disease using advanced ML, diverse data integration, and improved feature selection to boost accuracy and guide clinical decisions, ultimately improving patient outcomes. Shibata et al. [9] assessed and categorized the risk of major adverse cardiac events (MACEs) in patients with metabolic dysfunction-associated steatotic liver disease (MASLD) using a machine learning-based method. The study sought to identify key predictors of cardiac events, such as age and the count of metabolic risk factors, by applying a survival classification and regression tree (CART) model. Mohi Uddin et al. [10] presented a method for heart disease diagnosis using various ML techniques to predict and classify cardiac abnormalities, providing high accuracy.

Tsarapatsani et al. [11] predicted CVD mortality within ten years using clinical and biochemical data with various ML models. They evaluated the predictive accuracy of different models of ML, finding LR to be the most reliable. The results helped to estimate CVD risk scores for the TIMELY study. Furthermore, Gupta [12] determined the best ML model for diagnosing CVD from patients’ medical history. The study also aimed to reveal which of the supervised machine learning algorithms—decision tree, random forest, K-nearest neighbors, and support vector machine—supplied the highest level of accuracy, AUC, and F1-scores when compared to the other algorithms. As presented by Abhishek et al. [13], an ML model was developed to predict cardiovascular disease risk using benchmark datasets. The authors compared various imputation techniques and used the CatBoost algorithm, which achieved 91% accuracy with the Hungarian dataset, to improve prediction accuracy and healthcare outcomes. Nikam et al. [14] identified how body mass index (BMI) affects the prediction of cardiovascular disease using machine learning techniques. The study examined the role of BMI, among other features, in enhancing prediction accuracy through different regression and classification models.

As presented by Prakash et al. [15], a cardiac risk assessment system was designed using the LR classifier to assess the risk of CVD as accurately and clearly as possible. This research plan sought to show the appropriateness of this model, which was trained to 86.89% accuracy, enabling early detection of patients with CVD and aiding the prioritization of patients for a better healthcare trend. Dinesh et al. [16] generated a prognosis model for correctly assigning a given patient to the class of patients with some form of heart disease, and gave an awareness/diagnosis based on this assignment. The study aimed to increase the degree of prediction and apply and compare AWS ML algorithms with preprocessed medical data. The purpose was to enhance data analysis in decision-making for cardiovascular disease prediction and the performance of models. Jinjri et al. [17] identified the best ML model for helping to classify cardiovascular disease and determine whether patients have or do not have this disease. The paper assessed five ML classifiers to investigate which offered the best prediction model. The effectiveness of these models was assessed by comparing their accuracy in diagnosing cardiovascular disease using a dataset from the Kaggle repository, in order to determine the best approaches to cardiovascular disease diagnosis.

To identify the significant ensemble models of ML, Rath et al. [5] improved heart disease detection using machine learning, specifically focusing on ensemble models. They compared classifiers like AdaBoost and logistic regression (LR), and combined the best-performing models into an ensemble using majority voting. The ensemble model showed superior accuracy, F1-score, and AUC in detecting heart disease from ECG signals, highlighting its effectiveness and potential for broader applications in disease detection. Singh et al. [18] transformed ECG beat analysis into a fully automated classification process for normal and abnormal beat forms in order to detect arrhythmia using LSTM (long–short-term memory), and compared the results to the performance of other recurrent neural networks using the MIT-BIH arrhythmia database.

As presented in [20,21], advanced convolutional neural networks (CNNs) have been applied to classify ECG and detect arrhythmia. Mathunjwa et al. [19] constructed an extremely efficient algorithm to classify the various types of arrhythmia based on the emerging method of ECG RP for portable devices. The researchers improved classification performance by deploying a two-stage classification approach with the help of CNN classifiers, which were ResNet-18 and ResNet-50, to classify different arrhythmias. Zahid et al. [20] developed a new method for inter-patient ECG classification for the entire world to help capture arrhythmias, since both human and machine analyses of ECG signs face numerous difficulties. Proposing a compact in 1D Self-ONN architecture, this research added morphological and timing characteristics of heart cycles to the model, to improve the classification of arrhythmias. This approach aimed to achieve higher accuracy than other deep learning models, particularly for inter-patient classification based on ECG data.

Furthermore, Hannun et al. [6] applied deep neural networks (DNNs) to classify 12 different ECG rhythm classes using 91,232 single-lead ECGs from 53,549 patients. The DNNs demonstrated high performance, with an average ROC of 0.97 and an F1-score of 0.837, surpassing cardiologists in sensitivity and matching their specificity. These results indicate that deep learning could enhance the accuracy and efficiency of ECG analysis, potentially reducing misdiagnoses and improving the prioritization of critical cases in clinical practice.

Another deep learning model was presented in [21] for human activity classification with smart devices, where the size and complexity of the model, as well as several computations, were optimized to conserve battery power. This study compared handcrafted features to deep learning methods and proved that far fewer features can offer equally good performance. The method entailed transforming the IMU data into images of a two-dimensional plane, and opting for a concise CNN with an F1-score of 0.98. Also, to increase accuracy while decreasing memory and computational complexity, a hybrid SVM (support vector machine) was included. In addition, El Asnaoui [22] applied a single and ensemble deep learning model for classifying pneumonia using chest X-ray images. The study found that while InceptionResNet_V2 achieved an F1-score of 93.52% on its own, an ensemble model combining ResNet50, MobileNet_V2, and InceptionResNet_V2 reached a higher F1-score of 94.84%, indicating improved accuracy in pneumonia detection. In [23], a method for the classification of ECG signals was presented, which is patient-independent and implementable on any given monitoring device using DenseNet and GRU (gated recurrent unit) networks. Based on the MIT-BIH Arrhythmia and Supraventricular Databases, the models were found to have improvements of 10–12% with regard to F1-score, without the need to use the complex preprocessing WADB-UEC database. Smíšek et al. [24] developed another method for classifying ECG recordings into normal rhythm, atrial fibrillation, other rhythm, and noisy record classes. The approach used a two-step support vector machine (SVM) process: the first step involved creating a feature extraction of features of individual beats. Then, a second classification model was cultivated from these features. Ye et al. [25] developed a DNN model for classifying probabilities with heart rate variability (HRV), age, and sex, and introduced an ensemble classification strategy for cardiac arrhythmia detection. With this strategy, the average F1-score was 0.818. The study also assessed the importance of these variables in the Extreme Gradient Boosting (XGBoost) model to emphasize how explainable machine learning might improve the prediction of arrhythmias.

## 3. Methodology

### 3.1. Classification Using Full ECG Images

This part of our methodology builds on the foundational research we conducted, in which we examined different machine learning models for ECG image classification, obtaining an optimal F1-score of 96.5% through an ensemble approach that combined Inception, MobileNet, and NASNetLarge models. The research models that used complete ECG images including all 12 leads are the baseline for our ongoing investigation. A detailed review of whole ECG images enabled us to recognize the subtleties of cardiac conditions as they appear in the standard 12-lead format, which sets a superior benchmark for classification accuracy in heart disease.

### 3.2. Focusing on Individual ECG Leads

Informed by the success of our ensemble model utilizing full ECG images, our current research explores the diagnostic utility of images from individual ECG leads. This approach is driven by the potential to streamline the diagnostic process and enable real-time, continuous cardiac monitoring through wearable technology. Each ECG lead offers a distinct window into the heart’s electrical activity, providing critical information about specific cardiac regions and their associated conditions, as follows:

A. Limb Leads (I, II, III, aVR, aVL, aVF): These leads offer views of the heart’s electrical activity in the frontal plane, each highlighting different facets:
  - Lead I records the difference between the right and left arm electrodes, shedding light on the lateral wall of the heart.
  - Lead II measures the electrical activity between the right arm and left leg electrodes, and is particularly valuable for arrhythmia detection.
  - Lead III provides data from the difference between the left arm and left leg electrodes, crucial for identifying inferior myocardial infarctions.
  - The augmented limb leads (aVR, aVL, and aVF) supplement these perspectives by providing additional views that can help pinpoint specific cardiac issues, such as ischemia or injury.
B. Precordial Leads (V1-V6): These leads offer a horizontal plane view of the heart:
  - Lead V1 is positioned at the fourth intercostal space to the right of the sternum, and is instrumental in observing the right ventricle and septal region.
  - Lead V2 sits to the left of the sternum, mirroring V1’s view and aiding in diagnosing septal infarctions.
  - Lead V3 provides insight between V2 and V4, while V4, located at the midclavicular line on the fifth intercostal space, is key for anterior wall analysis.
  - Lead V5 and V6, aligned with the anterior axillary and midaxillary lines, respectively, are pivotal for assessing the lateral wall of the heart.

### 3.3. Proposal for Single-Lead-Based Model

Our study seeks to exploit the special diagnostic potential of individual ECG leads while building on the comprehensive anatomical understanding provided by the exact electrode placement of limb and precordial leads. By concentrating on one ECG lead, an original method that could revolutionize the monitoring and diagnosis of heart diseases is implemented. The developed model aims to pave the way for wearable devices that provide continuous, real-time monitoring of heart activity.

**Figure 1:**
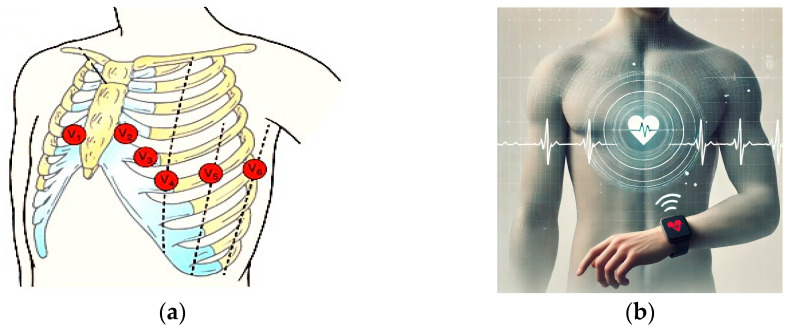
Electrode placement and single-lead ECG integration with wearable devices. (**a**) Electrode placement for precordial leads (V1 to V6) with red circles over the chest wall. (**b**) Single-lead ECG integration via wearables.

As shown in Figure 1, the electrode placement for lead V4 is derived from the precordial configuration, a key diagnostic lead in cardiac evaluations. Wearable devices, such as smartwatches or wireless chest patches, can leverage this lead’s data to deliver actionable health insights. These devices are designed to transmit ECG data wirelessly to cloud platforms or smartphones for further analysis using AI models.

This integration has several practical implications:

1. Early Diagnosis and Monitoring in Real Time: Wearable ECG devices provide users with a reliable means of detecting abnormalities like MI, PMI, and arrhythmias in their early stages. This is especially crucial for at-risk populations who require frequent monitoring.
2. Accessibility in Remote or Resource-Limited Settings: Single-lead ECG devices are portable, affordable, and do not require the infrastructure necessary for traditional multi-lead ECG setups, making them ideal for use in rural or underserved areas.
3. Enhanced Patient Engagement: The ability to monitor heart activity seamlessly in daily life through wearable technology can foster greater patient compliance and awareness, leading to improved overall cardiac health management.
4. Support for Telemedicine: Data collected by wearable devices can be integrated into telemedicine workflows, enabling healthcare providers to make informed decisions remotely, without requiring patients to visit clinics or hospitals.

This approach not only addresses a critical healthcare need, but also sets the foundation for scalable, AI-powered diagnostic solutions. Figure 1 illustrates the electrode placement for ECG leads, paired with a conceptual visualization of wearable ECG integration for continuous monitoring.

### 3.4. Dataset Description

For this study, we utilized a specialized dataset of ECG recordings [26], carefully compiled to support research endeavors in diagnosing and understanding cardiovascular diseases. This dataset stands as a pivotal resource for the medical research community, enabling the exploration and enhancement of diagnostic algorithms tailored to cardiac health assessment. It comprises ECG recordings that encapsulate a diverse range of cardiac anomalies, systematically categorized into four distinct groups for comprehensive analysis:

1. MI Patients: Included in this category are ECG recordings from individuals who have suffered from myocardial infarction. This condition, marked by the cessation of blood flow to a portion of the heart, leads to significant damage to the heart muscle.
2. Individuals with Arrhythmias: This group encompasses recordings from persons exhibiting irregular heart rhythms. Arrhythmias vary widely in their severity and implications, ranging from harmless to potentially fatal disorders.
3. Patients with Previous MI: This collection features ECG recordings from individuals with a history of myocardial infarction. Analyzing these recordings provides insights into the enduring impacts of MI on the heart, and aids in identifying markers for potential recurrence. The dataset includes only STEMI (ST-Elevation Myocardial Infarction) ECGs, with annotations specifying the affected myocardial wall, including anterior, inferior, lateral, and posterior walls. The superior performance of lead V4 in detecting myocardial infarctions can be attributed to its placement at the midclavicular line over the left ventricle, making it particularly sensitive to changes in the anterior wall, a region frequently affected in STEMI cases. This alignment provides a direct view of the electrical activity in the heart’s anterior region, enhancing the accuracy of diagnosis.
4. Control Group (Normal Individuals): This set includes recordings from individuals without any diagnosed cardiac conditions, serving as a baseline for normal heart functionality and rhythm. This comparison is essential for distinguishing between healthy and pathological heart conditions.

**Figure 2:**
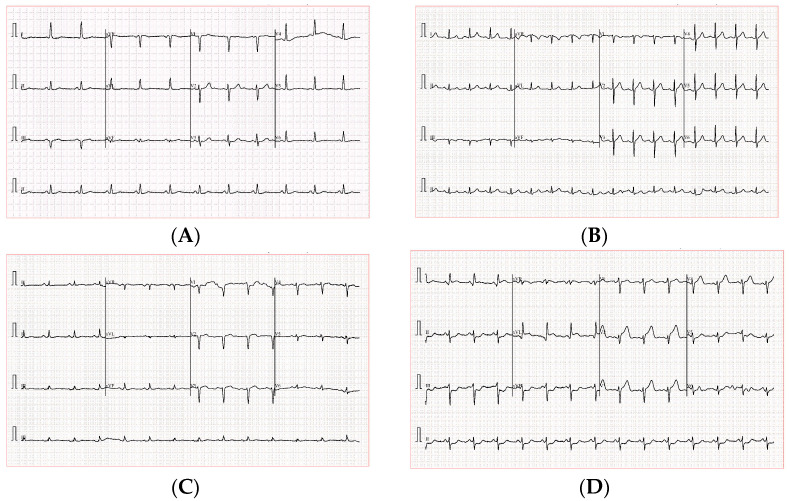
Sample ECG images for each cardiac condition in the dataset: (**A**) Normal, (**B**) Abnormal, (**C**) PMI, and (**D**) MI. These examples showcase the diverse signal patterns used for classification.

To provide a visual representation of the dataset, Figure 2 illustrates sample ECG images from each cardiac condition included in this study: Normal, Abnormal, PMI, and MI. These examples highlight the distinct signal patterns that are characteristic of each condition, which the deep learning models leverage to achieve accurate classification. The variations in waveform structure, amplitude, and rhythm emphasize the diagnostic value of ECG data in identifying cardiac abnormalities.

The objective of employing this dataset is to devise a classification model that is capable of not only distinguishing between healthy and unhealthy cardiac states, but also pinpointing specific heart diseases. Utilizing transfer learning techniques, the model aims to enhance the accuracy of early disease detection, thereby improving patient prognosis and contributing to cost-efficiency in healthcare. The dataset details, including the distribution of ECG recordings across the four categories, are summarized in the following text, providing a foundation for our analytical approach. In the dataset we used for our study, a total of 928 ECG recordings are systematically organized into four principal classes, to support a detailed examination of a variety of cardiac conditions. The dataset is made up of 284 recordings from individuals who are normal, acting as the control group to set a baseline for proper cardiac function. A total of 239 recordings illustrate the category of patients who have suffered an MI, showcasing cases in which the heart muscle has suffered damage from inadequate blood supply. In addition, the dataset has 172 recordings from people with a history of myocardial infarction (HMI), which provides understanding of the enduring effects and recurrent risks associated with MI. The fourth group includes 233 recordings from people presenting with abnormal heart rhythms or arrhythmias, which include both benign and severe conditions. This complete collection of ECG recordings supports our effort to create a detailed classification model that can tell apart healthy and pathological states, as well as multiple specific cardiac abnormalities.

### 3.5. Preprocessing of ECG Leads

**Figure 3:**
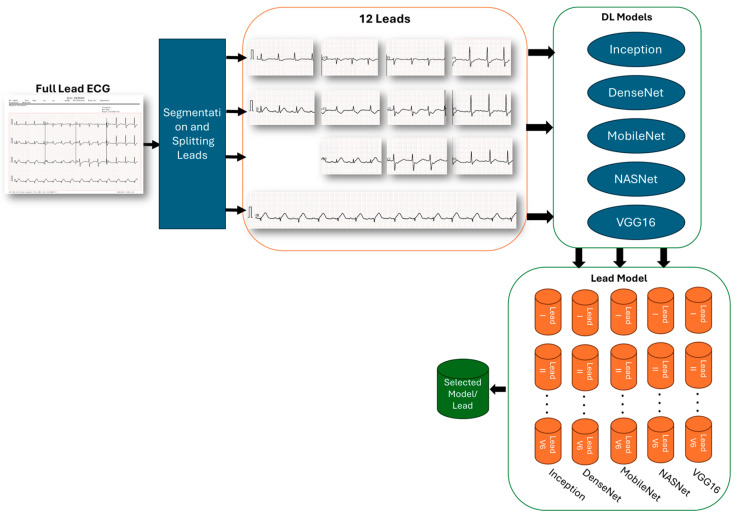
Framework for single ECG lead analysis using deep learning models for cardiac condition diagnosis.

An essential element of our research methodology was the preprocessing of ECG data, which guaranteed that the data entered into our models were of superior quality and consistent. The phase of preprocessing was focused on segmenting and separating the 12 individual lead traces from complete ECG recordings, as shown in the ECG report. The recordings at issue provided a complete image of the heart’s electrical activity from several anatomical perspectives, but for focused analysis and modeling, we needed to separate them into individual lead traces. Our method takes advantage of a detailed image splitting pipeline that emphasizes both precision and efficiency. Thanks to the standardized and normalized nature of the dataset we obtained, we could perform segmentation efficiently. Our system was skilled at precisely separating and extracting all 12 standard leads from the complete ECG report, as shown in Figure 3. The segmentation operation was followed by a systematic division of the ECG image into 12 discrete images, each representing a standard lead (I, II, III, aVR, aVL, aVF, V1, V2, V3, V4, V5, and V6). Subsequently, these images were standardized concerning size and resolution, promoting uniformity throughout the dataset—a critical factor for subsequent comparative analyses.

The culmination of this preprocessing phase was the production of a set of pristinely isolated lead-specific ECG images, exemplified in Figure 3, primed for the modeling phase. This meticulous process allows for the deployment of highly specialized diagnostic models, each tailored to detect and analyze the pathologies that are most prominently reflected in specific ECG leads. The described methodology not only enhances diagnostic acumen, but also emphasizes the immense value of individual lead data in achieving diagnostic precision in cardiac healthcare.

### 3.6. Integration of Deep Learning in Single-Lead ECG Diagnostics

We have reached an important milestone in cardiac diagnostics through machine learning with our framework, which takes advantage of deep learning to examine single-lead ECG data. The conceptual diagram in Figure 3 illustrates our comprehensive process: starting with the division and segmentation of a standard ECG into its separate leads, each lead independently feeds into a set of Deep Transfer Learning (DTL) models. This pipeline is designed to exploit the detailed data presented by each lead, enhancing a granular analysis that has not been explored before in assessments of cardiac health.

In our research, we focus on several DTL architectures that have consistently delivered promising results in image recognition tasks, including the interpretation of intricate patterns in ECG data.

These models are known for their excellence in various image recognition tasks for full-lead ECG data interpretation:

- Inception: Distinguished by its profound architecture that adeptly manages image recognition at scale.
- DenseNet201: Notable for its densely interconnected layers that foster feature retention and minimize parameter usage.
- VGG16: Esteemed for its straightforward yet deep framework that has demonstrated exceptional feature extraction capabilities.

In conjunction, we reintegrate the ensemble model that marked our previous success with full-lead ECG analysis, comprising the above-mentioned Inception in synergy with the following:

- MobileNetV2: Recognized for its compact architecture tailored for mobile deployment.
- NASNetLarge: Acclaimed for its adaptability and self-learning convolutional layer structure.

The selection of these models is based on their proven ability to balance performance and efficiency, making them well-suited for ECG analysis. Inception and DenseNet201 excel in extracting detailed features from complex data, which is crucial for detecting subtle patterns in ECG signals. VGG16, known for its simplicity and reliability, serves as a benchmark due to its established success in medical image processing. MobileNetV2 is particularly advantageous for its lightweight architecture, enabling real-time analysis in resource-constrained settings, such as wearable devices or telehealth platforms. NASNetLarge was chosen for its adaptability, leveraging neural architecture search to optimize performance across diverse datasets. Together, these models represent a diverse mix of architectural strengths, allowing for a comprehensive evaluation of single-lead ECG diagnostics across a range of use cases.

Our validation method is grounded in a stratified 5-fold cross-validation model, ensuring balanced class representation for robust model assessment. Each DTL model underwent rigorous training and evaluation on the isolated ECG leads, aiming to identify the most accurate diagnostic outcomes. The pipeline led to an extensive comparative analysis considering the following:

- Performance Metrics: Employing accuracy, precision, recall, and F1-score to assess model performance across cardiac conditions.
- Lead-Specific Performance: Analyzing how different leads perform to discern those with the highest diagnostic value for specific heart conditions.
- Efficiency Review: Exploring the balance between accuracy and prediction speed to determine the practicality of each lead as a potential solitary diagnostic tool.

This framework intends to expose the best synergy of models and leads that can most precisely diagnose cardiac conditions. The goal of the modeling phase was to discover the most effective combinations of leads and models for the accurate recognition of cardiac conditions. This project will both deepen our insight into ECG lead data and provide information for the creation of more accurate and efficient diagnostic solutions. Our target is to create a new standard for the use of AI-driven ECG analysis that is focused on leads in different medical settings, such as clinical environments and remote monitoring systems, to improve early intervention and patient care.

### 3.7. Experimental Setup

Our study into single-lead ECG diagnostic modeling includes an elaborate experiment setup supported by a strong mathematical framework. This project focuses on the evaluation of individual deep transfer learning models alongside each individual ECG lead. Stratified Cross-Validation for Model Fidelity: In order to maintain model accuracy and stability for a range of cardiac conditions, we implemented a stratified 5-fold cross-validation method. This approach secures class proportionality in every fold, which promotes a balanced and complete evaluation. Dynamic Training with Early Stopping: The training of models was both dynamic and iterative, using an early stopping mechanism optimized by F1-score. This brings the training process to a stop when the validation set performance reaches saturation point, which prevents overfitting and keeps computational efficiency intact.

Aggregated Validation for Comprehensive Assessment: Post-training, the predictions from each fold’s validation set were concatenated to form a unified dataset. This aggregated dataset facilitates a broad performance evaluation across the entire spectrum of the data, covering a range of conditions, from MI to normal cardiac rhythms. The culmination of the experiment presents us with a detailed analysis of the classification capability of each model when applied to individual ECG leads, setting a benchmark for selecting a suitable single lead for an identification task.

## 4. Result

Before diving into class-by-class nuances, we systematically examined each single ECG lead with five deep learning architectures—NASNetLarge, Inception, DenseNet201, MobileNetV2, and VGG16—to see how well they could classify Normal, Abnormal, PMI, and MI. This section covers lead-by-lead performance (Section 4.1), class-wise observations (Section 4.2), the accuracy vs. inference time trade-off (Section 4.3), key observations (Section 4.4), and an in-depth analysis of the optimal model–lead pairing (Section 4.5).

### 4.1. Lead-by-Lead Performance

**Figure 4:**
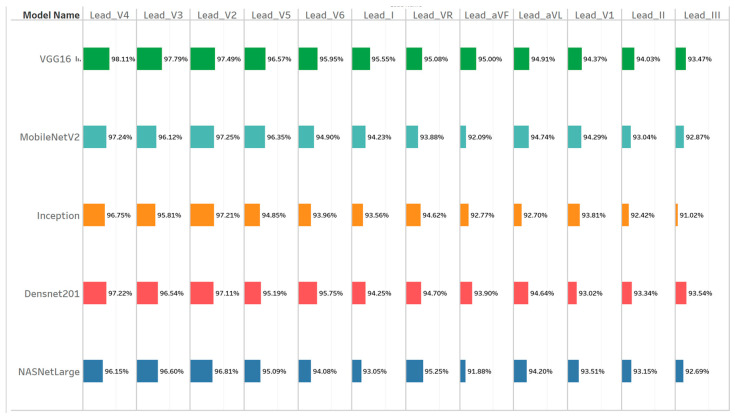
ECG model–lead analysis.

Our initial comparison focused on how effectively each of the five deep learning models performed on each single ECG lead (I, II, III, aVR, aVL, aVF, V1–V6). As shown in Figure 4, which visualizes the F1-scores for all model–lead pairings, lead V4 stands out, with F1-scores around 96–98% for most models, peaking at 98.11% (VGG16). Leads V2, V3, and V5 also frequently achieve high accuracy, typically falling in the 94–97% band. In contrast, the F1-scores for the limb leads (I, II, III, aVR, aVL, aVF) range between roughly 91 and 95%. For instance, the lowest F1 in the limb lead category appears when Inception tackles lead III (91.02%), whereas the best limb lead performance hits ~95% (e.g., VGG16 on aVR or aVF). For a numerical breakdown of precision, recall, and F1 for all model–lead combinations, Appendix A contains Comprehensive Performance Metrics Across ECG Leads tables.

A concise top five list, extracted from Figure 4, highlights the best single-lead performances overall, as shown in Table 1.

Notably, VGG16 and MobileNetV2 appear multiple times in this table, underscoring their effectiveness in single-lead ECG classification. DenseNet201 also ranks well when paired with V4, while Inception and NASNetLarge place just outside the top five.

### 4.2. Class-Wise Observations

**Figure 5:**
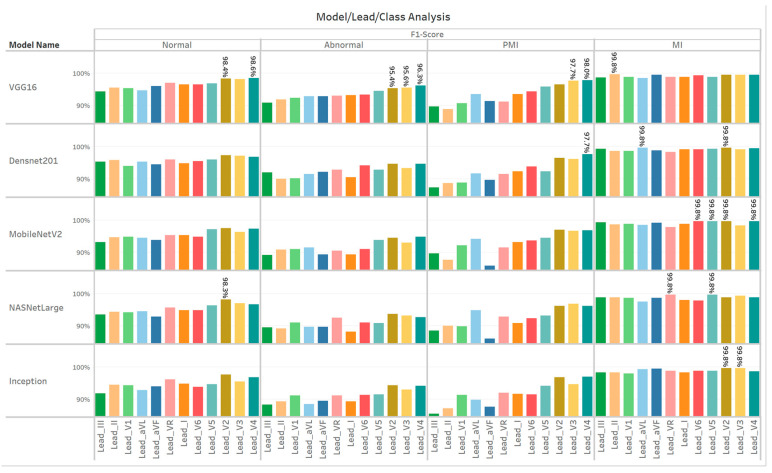
Analysis of ECG leads with cardiac models.

Having established a solid lead-by-lead picture in Section 4.1, we next turn to the class-wise outcomes. Figure 5 illustrates how each model–lead combination performed specifically for Normal, Abnormal, PMI, and MI. While different leads and models demonstrate varied strengths, updated analysis confirms that lead V4 dominated for most conditions, particularly when paired with VGG16. Meanwhile, MobileNetV2 excelled at detecting MI across multiple precordial leads.

Table 2 is a short summary table highlighting the top performer for each condition:

These findings underscore the particular diagnostic richness of V4 for most classes, where VGG16 surpasses even other high-performing models. For MI, however, MobileNetV2 achieves a near-perfect 99.8% F1 on several precordial leads (V2, V4, V5, V6), showcasing its adaptability. Taken as a whole, these results strongly support the feasibility of single-lead ECG setups, especially from V4, for reliable detection of multiple cardiac conditions.

The Comprehensive Performance Metrics Across ECG Leads tables in Appendix A likewise confirm minimal confusion between the Normal and MI categories, though there is some moderate overlap between Abnormal and PMI in a few leads.

### 4.3. Accuracy vs. Inference Time

**Figure 6:**
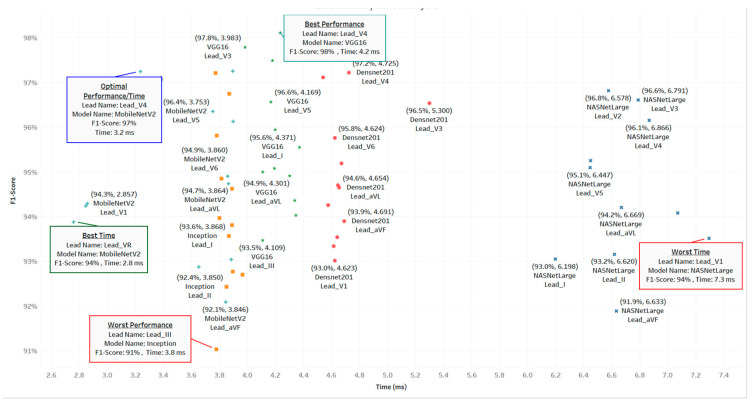
Performance/time analysis for ECG models.

In practical deployments, clinicians and developers often balance classification accuracy with prediction speed. Figure 6 plots each model–lead combination according to F1-score (vertical) and inference time (horizontal). There are five noteworthy points from Figure 6:

- Lead V4, VGG16: best performance at 98.11% F1, with a modest 4.2 ms latency.
- Lead V4, MobileNetV2: optimal performance/time synergy at 97.24% F1, 3.2 ms.
- Lead VR, MobileNetV2: best time (2.8 ms) at 93.88% F1.
- Lead III, Inception: worst performance at 91.02% F1 and ~3.8 ms.
- Lead V1, NASNetLarge: worst time (7.3 ms), albeit ~93.51% F1.

This distribution signals that MobileNetV2 generally offers the top speed–accuracy balance, while VGG16 is favored where absolute F1 is paramount. NASNetLarge, although powerful, is hampered by increased inference times, and Inception occasionally lags in F1, despite quicker runs.

### 4.4. Key Observations

A single-lead ECG, particularly with lead V4, can feasibly match multi-lead accuracy without the overhead of a full 12-lead array. VGG16, at about 138 million parameters (roughly 530–550 MB in float32 format), nearly always captures the highest F1-scores (98.11% on V4), despite a moderately higher inference time. This trade-off between size and latency can be acceptable in clinical settings, where absolute performance outweighs memory constraints.

On the other hand, MobileNetV2 occupies a far smaller footprint (~3.4 million parameters, or 12–15 MB), loses little accuracy (~97.24% F1 on V4), and accelerates inference to ~3.2 ms. This markedly compact model is thus highly appealing for real-time or resource-constrained environments, including wearables or remote triage systems, where both speed and memory efficiency matter. MobileNetV2’s efficiency and compact size (~13 MB, 3.2 ms inference time) also make it an ideal candidate for integration into advanced medical devices such as electronic stethoscopes. This would enable real-time, automatic diagnoses of cardiac conditions, improving accessibility and early detection, particularly in remote or resource-limited settings.

### 4.5. In-Depth Analysis of the Optimal Model–Lead Pairing

**Figure 7:**
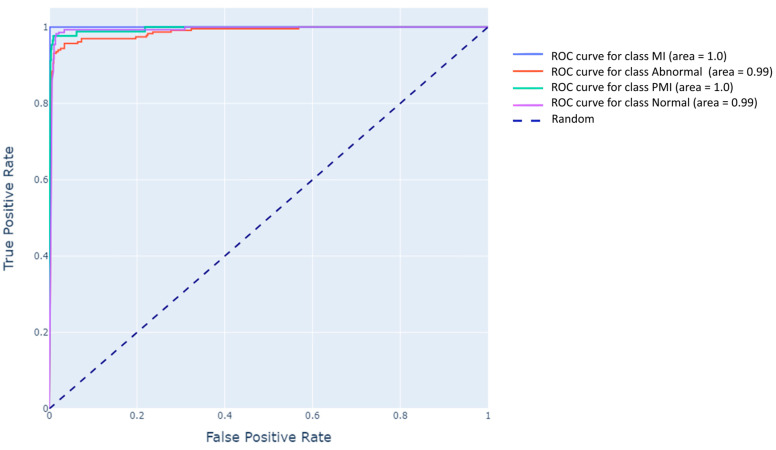
Optimal model/lead performance analysis.

As we delve into the final section of our analysis, we concentrate on the optimal model–lead combination identified from previous comparisons: MobileNetV2 using lead V4. A detailed evaluation of its diagnostic capabilities is illustrated by the Receiver Operating Characteristic (ROC) curves in Figure 7, and by a confusion matrix summarizing classification outcomes across Normal, Abnormal, PMI, and MI.

Figure 7 (MobileNetV2 + lead V4 ROC Curves) underscores the model’s outstanding performance across all classes:

- MI: The area under the ROC curve achieves an AUC of 1.00, showing that the model can distinguish MI from other traces with 100% accuracy.
- Abnormal Heartbeat: The ROC curve yields an AUC of 0.99, reflecting high sensitivity and specificity for arrhythmic patterns.
- Previous MI (PMI): Once more, the AUC is a flawless 1.00, capturing the subtle lingering effects of past infarction.
- Normal Heart Function: The AUC stands at 0.99, confirming that the model reliably differentiates healthy ECG signals from pathological ones.

These curves emphasize that MobileNetV2 + lead V4 attains near-perfect classification, with minimal false positives for each class. Complementing the ROC findings, Table 3 presents the confusion matrix for the same model–lead pairing:

According to Table 3, the model achieves 100% accuracy for MI, with no false positives or negatives. Abnormal heartbeats are classified correctly 93% of the time, with minor misclassifications into Normal (3.5%), PMI (3%), and MI (0.5%). PMI achieves a strong detection rate of 98%, and Normal function is also correctly identified 98% of the time. While slight misclassifications exist, their negligible clinical impact underscores the model’s overall robustness and reliability.

By merging the ROC curves and confusion matrix data, we obtain a comprehensive view of how proficiently MobileNetV2 + lead V4 discerns each cardiac condition. From a clinical perspective, this model–lead combination forms a reliable, accurate, and time-efficient diagnostic tool, which is particularly vital for automated systems, telehealth platforms, and wearable devices demanding near-real-time ECG analysis. Its capacity to differentiate between normal and pathological signals with such precision can streamline triage, sharpen treatment strategies, and ultimately elevate patient outcomes.

Furthermore, considering MobileNetV2’s lightweight architecture (~3.4 million parameters, ~12–15 MB on disk), alongside its short inference time (~3.2 ms), we see strong potential for broad clinical deployment. Not only does the model satisfy high accuracy standards, but it also sets a benchmark for future AI-assisted cardiology solutions aimed at early detection and timely intervention in both acute and chronic cardiac conditions.

## 5. Discussion

This study underscores the transformative potential of deep learning models in leveraging single-lead ECG data for cardiac diagnostics. Through a meticulous evaluation of multiple architectures and ECG leads, the findings reveal a balance between accuracy and computational efficiency, offering insights into their suitability for diverse healthcare applications. However, this exploration also sheds light on key trade-offs and challenges associated with these approaches.

### 5.1. Trade-Offs Between Model Complexity and Performance

The analysis reveals that while models like VGG16 achieve the highest F1-scores (98.11% with lead V4), their relatively larger size (~528 MB) and inference time (4.2 ms) present challenges in resource-constrained settings. On the other hand, MobileNetV2, with its smaller size (~13 MB) and faster inference time (3.2 ms), emerges as a practical alternative for real-time monitoring and wearable technologies. This trade-off between model complexity and performance emphasizes the importance of tailoring model selection to specific use cases.

The performance differences observed between models stem from their distinct architectural characteristics. VGG16, with its deep sequential structure and extensive parameter count (~138 M), excels in feature extraction, but comes at the cost of higher computational demands. This is reflected in its strong F1-score (98.11%) but relatively large size (~528 MB), making it ideal for high-precision tasks, but less practical for real-time deployment.

MobileNetV2, in contrast, utilizes depth-wise separable convolutions, significantly reducing computational complexity while maintaining competitive performance (97.24% F1-score). Its lightweight architecture (~13 MB) and faster inference time (3.2 ms) make it a strong candidate for telehealth applications and wearable ECG monitoring.

Inception and DenseNet201, known for their multi-path feature extraction and densely connected layers, provide efficient feature retention, but require more processing power. NASNetLarge, despite its adaptability via neural architecture search, does not outperform VGG16 and MobileNetV2, likely due to overfitting tendencies when applied to single-lead ECG data.

These insights underscore the need for selecting models based on not just accuracy, but also computational efficiency, particularly when considering deployment in resource-constrained environments. Additionally, ensemble deep learning approaches have demonstrated significant potential in cardiac diagnostics by leveraging the strengths of multiple architectures for precise ECG classification. Such methods can offer enhanced accuracy, but may introduce additional computational overhead, requiring careful consideration in practical applications [27], such as in the following scenarios:

1. High-Accuracy Scenarios: In critical clinical settings where accuracy is paramount, models like VGG16 are more appropriate, due to their superior diagnostic capability.
2. Resource-Constrained Scenarios: For telehealth or wearable applications, MobileNetV2 strikes an optimal balance, offering near-competitive accuracy with significantly lower computational requirements.

This dual perspective highlights the importance of context-driven model deployment, acknowledging that the “best” model is not universally applicable, but varies based on the intended application.

This study is the first to systematically evaluate single-lead ECG data using advanced deep learning models. By leveraging single-lead data, our approach eliminates the need for complex multi-lead systems, making cardiac diagnostics more portable and scalable. This advancement bridges a critical gap in the literature and paves the way for deploying lightweight diagnostic solutions in resource-constrained and remote environments.

To further analyze the learning behavior of VGG16 and MobileNetV2 with lead V4, we plotted the training and validation accuracy/loss curves across five-fold cross-validation. These curves, presented in the Appendix B, demonstrate convergence within 100 epochs, with early stopping applied to prevent overfitting. The plots highlight the consistent convergence and stability of both models, with MobileNetV2 showing slightly faster convergence due to its lightweight architecture. This analysis reinforces the robustness of both models for single-lead ECG diagnostics across various scenarios.

### 5.2. Challenges in Clinical Deployment

Deploying these models in real-world clinical settings introduces several challenges that must be addressed. While the use of single-lead ECG data simplifies the diagnostic process and reduces dependency on complex equipment, several challenges remain for clinical deployment. These include ensuring generalizability to diverse patient populations, maintaining diagnostic accuracy in noisy or variable conditions, and integrating the models seamlessly into existing workflows:

1. Computational Limitations: While lightweight models like MobileNetV2 are feasible for wearable devices, deploying larger models such as VGG16 in embedded systems may require hardware upgrades or cloud-based processing, which could increase latency.
2. Data Variability: Real-world ECG signals, often captured in noisy environments or from diverse populations, may deviate from the curated dataset used in this study. Models must be robust to handle such variability without compromising accuracy.
3. Interpretability: Clinicians may require additional tools or visualizations to interpret the predictions of deep learning models effectively, particularly for high-stakes decisions.
4. Regulatory Approval: Ensuring compliance with regulatory standards for medical devices is another hurdle that could delay model deployment.

### 5.3. Limitations

While the findings of this study are promising, certain limitations warrant attention:

1. Dataset Bias: The dataset used in this study is derived from a specific population, which may introduce biases that limit the generalizability of the results to broader demographics.
2. Static Data: The reliance on static ECG images rather than dynamic signals constrains the applicability of these models to real-time scenarios. Future work could focus on incorporating temporal information for enhanced diagnostic accuracy.
3. Scalability: The study primarily evaluates single-lead ECG data, representing a novel contribution to the literature. While this approach introduces a significant advancement in portability and accessibility, multi-lead data often provide complementary diagnostic insights, which this work does not explore. Future research could investigate hybrid approaches combining single- and multi-lead data to further enhance diagnostic precision.

By acknowledging these limitations, this study sets a foundation for future research, aiming to improve the robustness, scalability, and generalizability of single-lead ECG models. Overcoming these challenges will be critical for translating the potential of deep learning into tangible clinical benefits.

### 5.4. Future Directions

Future studies could focus on addressing these limitations by incorporating larger, more diverse datasets, exploring real-time ECG signals, and enhancing model interpretability. Additionally, hybrid approaches combining single-lead analysis with temporal data, or integrating lightweight models into wearable technology, could further refine diagnostic accuracy while maintaining computational efficiency. Through continued innovation and validation, single-lead ECG analysis has the potential to redefine cardiac diagnostics, making advanced healthcare accessible to resource-limited settings worldwide.

## 6. Conclusions

The integration of machine learning with ECG analysis has opened new opportunities for rapid and accurate cardiac diagnosis. This study bridges a crucial gap by exploring the diagnostic potential of deep learning models applied to single-lead ECG data, demonstrating their value beyond traditional multi-lead approaches.

Our analysis highlights VGG16 paired with lead V4 as a top performer, achieving an F1-score of 98.11%, with an inference time of 4.2 ms and a model size of ~530 MB. While its larger size may limit scalability, its precision makes it suitable for critical clinical settings. In contrast, MobileNetV2, achieving an F1-score of 97.24%, with a compact size (~12 MB) and inference time of 3.2 ms, provides an efficient alternative for real-time applications such as wearable devices and telehealth platforms.

These findings have significant implications for resource-constrained environments and scenarios requiring immediate diagnosis. MobileNetV2’s lightweight architecture enables broader accessibility to advanced ECG diagnostics, facilitating the deployment of affordable, scalable solutions. Both models can enhance clinical workflows, reduce the burden on healthcare professionals, and foster patient engagement through wearable technologies.

In conclusion, single-lead ECG analysis powered by deep learning offers accessible, efficient, and reliable alternatives to multi-lead methods, signaling a paradigm shift in cardiac healthcare. Future efforts should focus on optimizing these models for interpretability and generalizability across diverse populations. AI-driven single-lead ECG diagnostics have the potential to revolutionize cardiac care, making advanced diagnostics more accessible worldwide and transforming how cardiovascular diseases are managed.

## Figures and Tables

**Table 1:** Top five model–lead performances.

Rank	Model–Lead	F1-Score
1	VGG16 + V4	98.11%
2	VGG16 + V3	97.79%
3	VGG16 + V2	97.49%
4	MobileNetV2 + V2	97.25%
5	MobileNetV2 + V4	97.24%

**Table 2:** Summary of best model–lead performance per class.

Class	Model–Lead Pair	F1-Score
Normal	VGG16 + V4	98.6%
Abnormal	VGG16 + V4	96.3%
PMI	VGG16 + V4	98%
MI	MobileNetV2 + (V2, V4, V5, or V6)	99.8%

**Table 3:** Confusion matrix.

	Myocardial Infarction (MI)	Abnormal	Previous Myocardial Infarction (PMI)	Normal
Myocardial Infarction (MI)	100%	0%	0%	0%
Abnormal	0.5%	93%	3%	3.5%
Previous Myocardial Infarction (PMI)	0%	1%	98%	1%
Normal	0%	2%	0%	98%

**Figure A1:**
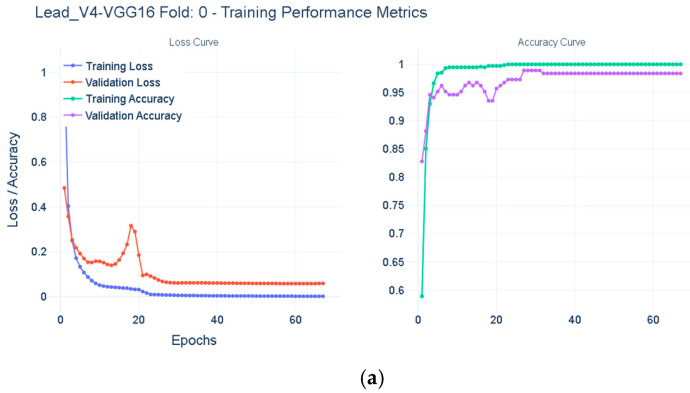

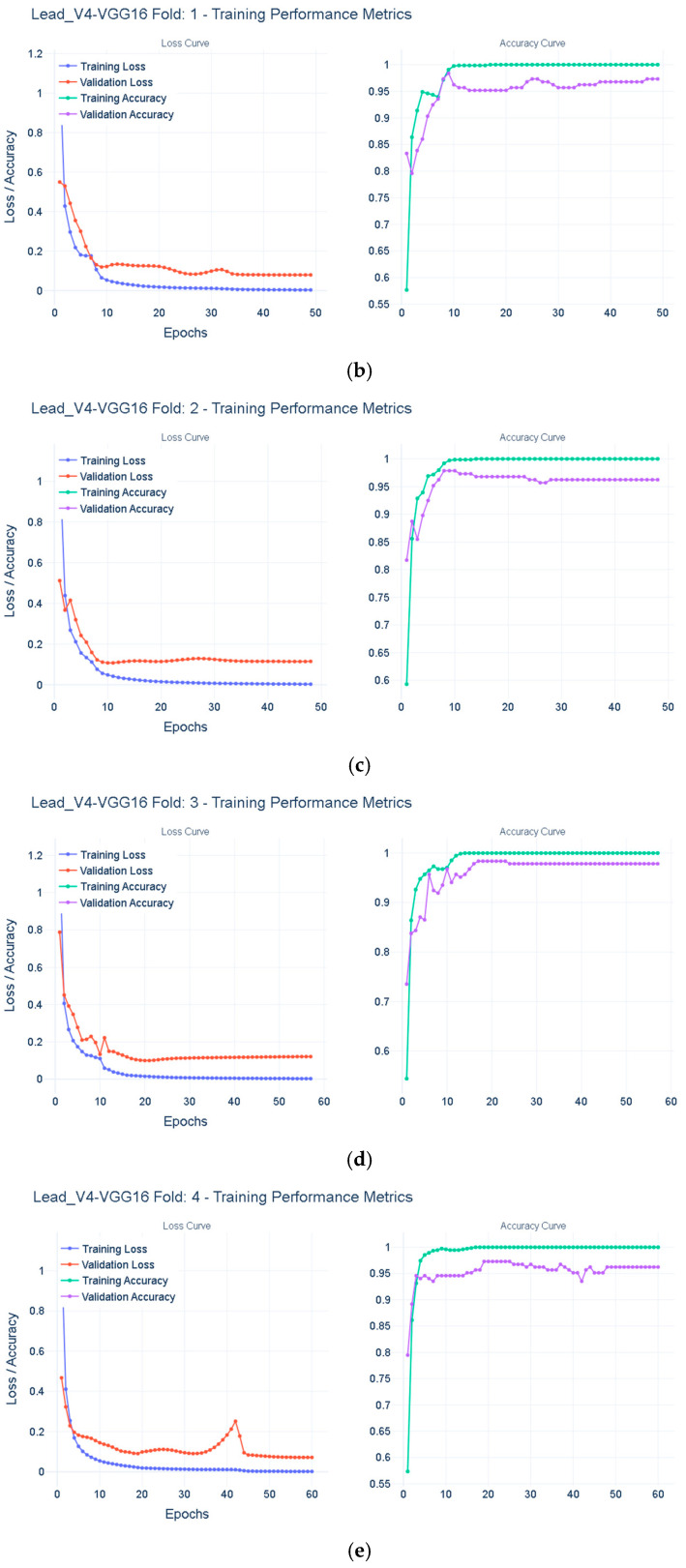
Training curves for VGG16 with lead V4. These curves demonstrate consistent convergence within 100 epochs, with early stopping ensuring efficient training. (**a**) The training and validation accuracy/loss curves for VGG16 with lead V4 across fold 0. (**b**) The training and validation accuracy/loss curves for VGG16 with lead V4 across fold 1. (**c**) The training and validation accuracy/loss curves for VGG16 with lead V4 across fold 2. (**d**) The training and validation accuracy/loss curves for VGG16 with lead V4 across fold 3. (**e**) The training and validation accuracy/loss curves for VGG16 with lead V4 across fold 4.

**Figure A2:**
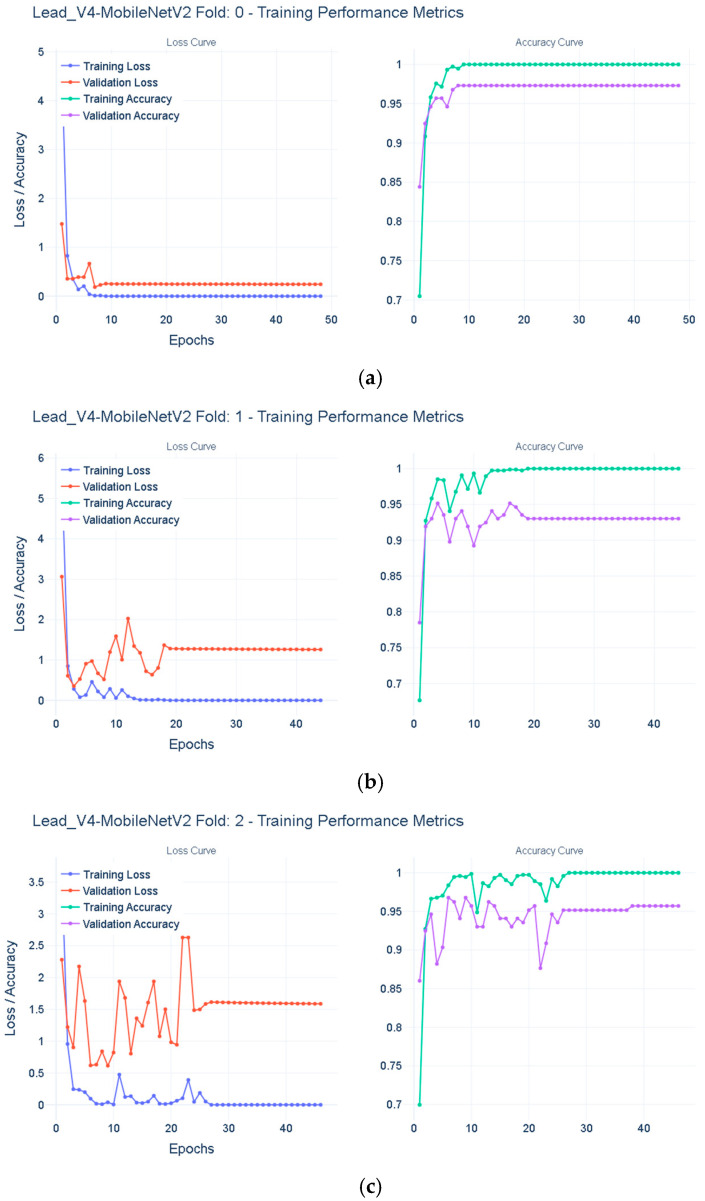

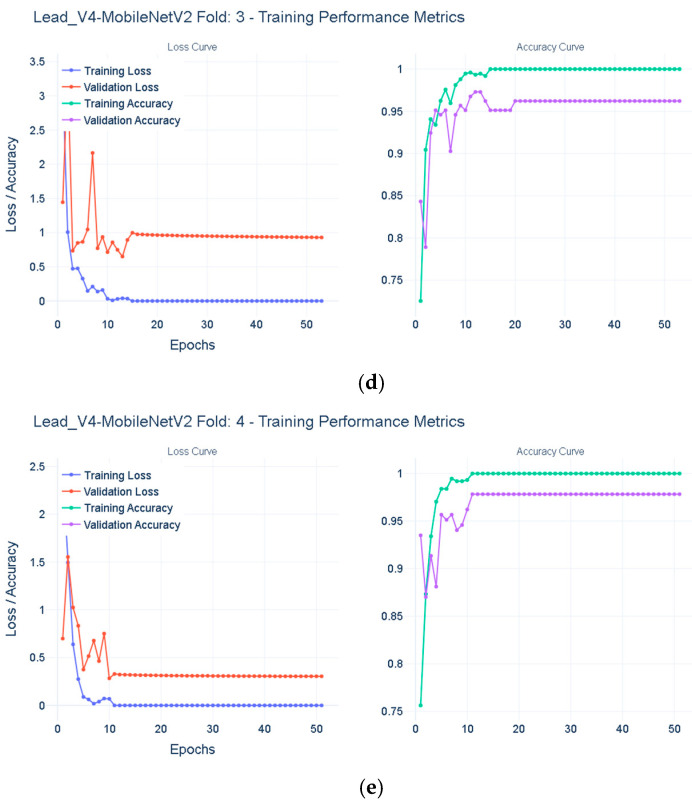
Training curves for MobileNetV2 with lead V4. These curves highlight MobileNetV2’s lightweight design, which enables slightly faster convergence, while maintaining high accuracy and stability across folds. (**a**) The training and validation accuracy/loss curves for MobileNetV2 with lead V4 across fold 0. (**b**) The training and validation accuracy/loss curves for MobileNetV2 with lead V4 across fold 1. (**c**) The training and validation accuracy/loss curves for MobileNetV2 with lead V4 across fold 2. (**d**) The training and validation accuracy/loss curves for MobileNetV2 with lead V4 across fold 3. (**e**) The training and validation accuracy/loss curves for MobileNetV2 with lead V4 across fold 4.

## Data Availability

The experimental data and the simulation results that support the findings of this study are available with the following identifier: https://data.mendeley.com/datasets/gwbz3fsgp8/2 (accessed on 30 January 2024) doi: 10.17632/gwbz3fsgp8.2.

## Appendix A. Comprehensive Performance Metrics Across ECG Leads

This section provides an overview of the appendix’s purpose. It details the performance of deep learning models applied to various ECG leads, with a focus on metrics like F1-score, inference time, precision, and recall. The data presented here support and extend the findings in the main paper.

### Appendix A.1. Performance Evaluation for Lead I

Lead I is one of the limb leads in an ECG. It is essential for offering understanding of the heart’s lateral wall, and is usually used for evaluating cardiac rhythms and recognizing pathological conditions. Because of its position across the arms, it delivers a unique view of the heart’s electrical signals, which is important for the early detection and diagnosis of a range of heart problems. Within the scope of Experiment 1, the evaluation of the models included precision, recall, and F1-score for classifying cardiac conditions from lead I. The performance of each model, as outlined in the results provided in Table A1, is summarized as follows:

**Table A1:** Performance on lead I.

Performance	Model Name	Lead I				Mean
		Normal	Abnormal	PMI	MI	
Precision	NASNetLarge	93.81%	95.96%	86.39%	96.37%	93.13%
	Inception	92.62%	92.24%	93.90%	96.76%	93.88%
	MobileNetV2	92.41%	96.98%	90.66%	97.95%	94.50%
	Densnet201	91.78%	95.26%	92.94%	98.35%	94.58%
	VGG16	94.90%	99.03%	90.71%	97.95%	95.65%
Recall	NASNetLarge	96.13%	81.55%	95.93%	100.00%	93.40%
	Inception	97.18%	86.70%	89.53%	100.00%	93.35%
	MobileNetV2	98.59%	82.83%	95.93%	100.00%	94.34%
	Densnet201	98.24%	86.27%	91.86%	100.00%	94.09%
	VGG16	98.24%	87.98%	96.51%	100.00%	95.68%
F1-Score	NASNetLarge	94.96%	88.17%	90.91%	98.15%	93.05%
	Inception	94.85%	89.38%	91.67%	98.35%	93.56%
	MobileNetV2	94.90%	90.54%	92.40%	99.17%	94.25%
	Densnet201	95.40%	89.35%	93.22%	98.96%	94.23%
	VGG16	96.54%	93.18%	93.52%	98.96%	95.55%

To explore the major performance of the deep learning models, we can conclude the results of lead I as follows:

- Precision: The models showcased robust precision in detecting the four cardiac conditions. VGG16 excelled, leading with a mean precision of 95.65%, indicating its precision in identifying true positive cases across all cardiac events. MobileNetV2 and DenseNet201 closely followed, showcasing their effective distinction between the conditions.
- Recall: Regarding recall, VGG16 and MobileNetV2 displayed exemplary performance, with mean recalls of 95.68% and 94.34%, respectively, highlighting their ability to identify the majority of actual cases.
- F1-Score: The F1-score, which balances precision and recall, is essential for gauging a model’s overall accuracy. VGG16 achieved a mean F1-score of 95.55%, reinforcing its efficacy in providing reliable detection across cardiac conditions. DenseNet201 also demonstrated a strong F1-score, indicating a well-balanced model.

The examination of lead I reveals that VGG16 stands out with high precision and recall, while DenseNet201 and MobileNetV2 also show substantial overall performance. The consistency of VGG16 in achieving high scores across precision, recall, and F1-score makes it a promising model for clinical application, specifically when utilizing lead I of the ECG for cardiac condition assessment.

### Appendix A.2. Performance Evaluation for Lead II

Lead II is one of the fundamental leads in the 12-lead ECG, and is particularly significant for evaluating the heart’s rhythm and identifying arrhythmias. It provides a clear view of the P wave, and is thus instrumental in diagnosing atrial abnormalities. Lead II’s angle of orientation makes it sensitive to the electrical activity of both the atria and ventricles, often making it the lead of choice in routine ECG monitoring. The performance metrics for lead II have been thoroughly evaluated for the same five deep learning models. A summary of the results is recorded in Table A2.

**Table A2:** Performance on lead II.

Performance	Model Name	Lead I				Mean
		Normal	Abnormal	PMI	MI	
Precision	NASNetLarge	91.69%	93.43%	90.59%	97.95%	93.42%
	Inception	92.88%	93.46%	87.21%	96.76%	92.58%
	MobileNetV2	94.72%	91.34%	88.24%	97.94%	93.06%
	Densnet201	94.52%	95.65%	85.87%	97.55%	93.40%
	VGG16	93.88%	94.14%	88.95%	99.58%	94.14%
Recall	NASNetLarge	97.18%	85.41%	89.53%	100.00%	93.03%
	Inception	96.48%	85.84%	87.21%	100.00%	92.38%
	MobileNetV2	94.72%	90.56%	87.21%	99.58%	93.02%
	Densnet201	97.18%	84.98%	91.86%	100.00%	93.51%
	VGG16	97.18%	89.70%	88.95%	100.00%	93.96%
F1-Score	NASNetLarge	94.36%	89.24%	90.06%	98.96%	93.15%
	Inception	94.65%	89.49%	87.21%	98.35%	92.42%
	MobileNetV2	94.72%	90.95%	87.72%	98.76%	93.04%
	Densnet201	95.83%	90.00%	88.76%	98.76%	93.34%
	VGG16	95.50%	91.87%	88.95%	99.79%	94.03%

To explore the major performance of the deep learning models, we can conclude the results of lead II as follows:

- Precision: The models showed a high degree of precision, with VGG16 exhibiting the highest mean precision at 94.14%, suggesting its strong capability in accurately classifying true positives for cardiac events as detected in lead II. DenseNet201 and NASNetLarge also displayed high precision, indicative of their effectiveness in differentiating specific heart conditions.
- Recall: For recall, VGG16 and DenseNet201 shared the highest mean scores of 93.96% and 93.51%, respectively, pointing to their considerable sensitivity in identifying actual positive cases for the conditions represented by lead II.
- F1-Score: When considering the F1-score, VGG16 achieved a top mean score of 94.03%, highlighting its balanced approach in precision and recall. DenseNet201 closely followed, with its F1-score reflecting a model well-tuned for accurate detection of cardiac anomalies in this lead.

The analysis of lead II suggests VGG16 stands out as a model well-equipped for clinical scenarios, with its high precision and recall indicating its potential as a reliable tool for cardiac diagnosis using this specific ECG lead. DenseNet201 and NASNetLarge also prove to be strong contenders, emphasizing the effectiveness of these models in clinical diagnosis, based on their performance metrics.

### Appendix A.3. Performance Evaluation for Lead III

Lead III is an arm lead on the ECG. It is important for evaluating the heart’s inferior part. It measures the electrical activity between the left arm and the left leg, delivering important data that can be particularly telling of inferior myocardial infarction and other associated conditions. The interpretation of lead III data for effective clinical diagnosis depends critically on the model’s accuracy, given its specific viewpoint on cardiac electrical activity. A detailed evaluation of the deep learning models on lead III yielded the results shown in Table A3.

**Table A3:** Performance on lead III.

Performance	Model Name	Lead I				Mean
		Normal	Abnormal	PMI	MI	
Precision	NASNetLarge	91.86%	92.27%	89.35%	97.95%	92.86%
	Inception	92.50%	92.49%	81.91%	96.76%	90.92%
	MobileNetV2	89.61%	91.82%	93.67%	98.76%	93.46%
	Densnet201	95.42%	94.98%	84.70%	98.76%	93.46%
	VGG16	91.69%	93.64%	92.59%	97.55%	93.87%
Recall	NASNetLarge	95.42%	87.12%	87.79%	100.00%	92.58%
	Inception	91.20%	84.55%	89.53%	100.00%	91.32%
	MobileNetV2	97.18%	86.70%	86.05%	100.00%	92.48%
	Densnet201	95.42%	89.27%	90.12%	100.00%	93.70%
	VGG16	97.18%	88.41%	87.21%	100.00%	93.20%
F1-Score	NASNetLarge	91.84%	88.34%	85.56%	98.35%	91.02%
	Inception	93.61%	89.62%	88.56%	98.96%	92.69%
	MobileNetV2	94.36%	90.95%	89.82%	98.76%	93.47%
	Densnet201	93.24%	89.18%	89.70%	99.38%	92.87%
	VGG16	95.42%	92.04%	87.32%	99.38%	93.54%

To explore the major performance of the deep learning models, we can conclude the results of lead III as follows:

- Precision: The precision across the models remained high. VGG16 led with a mean precision of 93.87%, showcasing its robust capability to accurately identify true positive cases for cardiac conditions as detected in lead III. DenseNet201 and MobileNetV2 both followed closely, exhibited mean precision rates of 93.46%, indicating strong model performance in classifying true positives within the context of lead III.
- Recall: In recall, VGG16 and DenseNet201 showed remarkable performance, with mean recall rates of 93.20% and 93.70%, respectively. These scores indicate that these models are highly capable of capturing the majority of actual positive cases, which is essential for clinical applications, where failing to detect a condition can be critical.
- F1-Score: The F1-score reflects the balance between precision and recall. Here, VGG16 stood out with a mean F1-score of 93.54%, underscoring its balanced classification capability. MobileNetV2 also displayed a high mean F1-score of 93.47%, pointing toward a well-rounded model that is adept at both identifying and classifying cardiac conditions accurately in lead III.

The assessment of lead III underscores the robustness of the evaluated models, particularly the VGG16, DenseNet201, and MobileNetV2, which all demonstrated significant capabilities in processing ECG data from this lead. The high precision and recall rates, along with strong F1-scores, reinforce the potential of these models to serve as reliable tools for diagnosing cardiac conditions through lead III analysis.

### Appendix A.4. Performance Evaluation for Lead aVR

As one of the standard limb leads in an electrocardiogram, lead aVR provides an exclusive angle on the heart’s electrical activity, from the right shoulder to the center of the heart. This lead is especially useful in the diagnosis of particular cardiac arrhythmias and myocardial ischemia. Accuracy in the analysis of aVR data is important for finding particular patterns that may reveal underlying heart conditions. We carried out extensive evaluation of the performance of our selected deep learning models on lead aVR, calculating precision, recall, and F1-scores for every model across a range of cardiac conditions. The summarized results are displayed in Table A4, as follows:

**Table A4:** Performance on lead aVR.

Performance	Model Name	Lead I				Mean
		Normal	Abnormal	PMI	MI	
Precision	NASNetLarge	93.90%	97.62%	90.16%	99.58%	95.32%
	Inception	95.17%	96.19%	89.13%	97.95%	94.61%
	MobileNetV2	95.74%	95.26%	88.17%	95.98%	93.79%
	Densnet201	94.24%	97.18%	91.33%	96.76%	94.88%
	VGG16	96.52%	96.76%	88.95%	97.95%	95.04%
Recall	NASNetLarge	97.54%	87.98%	95.93%	100.00%	95.36%
	Inception	97.18%	86.70%	95.35%	100.00%	94.81%
	MobileNetV2	95.07%	86.27%	95.35%	100.00%	94.17%
	Densnet201	97.89%	88.84%	91.86%	100.00%	94.65%
	VGG16	97.54%	89.70%	93.60%	100.00%	95.21%
F1-Score	NASNetLarge	95.68%	92.55%	92.96%	99.79%	95.25%
	Inception	96.17%	91.20%	92.13%	98.96%	94.62%
	MobileNetV2	95.41%	90.54%	91.62%	97.95%	93.88%
	Densnet201	96.03%	92.83%	91.59%	98.35%	94.70%
	VGG16	97.02%	93.10%	91.22%	98.96%	95.08%

To explore the major performance of the deep learning models, we can conclude the results of lead aVR as follows:

- Precision: In precision, NASNetLarge emerged as the top performer with 95.32%, demonstrating exceptional accuracy in identifying true positives. VGG16 also displayed remarkable precision at 95.04%, and Densnet201 closely followed with a strong mean precision of 94.88%. These figures suggest that the models have a high ability to correctly label the presence of cardiac events when analyzing lead aVR.
- Recall: For recall, VGG16 and NASNetLarge both displayed excellent performance, with mean recall rates of 95.21% and 95.36%, respectively. These high recall values indicate that these models are reliable for identifying the majority of true positive cases, which is critical in preventing misdiagnosis of cardiac conditions.
- F1-Score: When considering the F1-score, a measure that provides a balance between precision and recall, NASNetLarge stood out with a mean F1-score of 95.25%. VGG16 followed with a mean F1-score of 95.08%, underscoring its effective balance in detecting true positives while minimizing false negatives and false positives. These high F1-scores suggest that these models are adept at providing reliable and accurate diagnoses using lead aVR.

These measures show the possible usefulness of using these deep learning models in clinical environments where lead aVR is employed for cardiac evaluation. The superior performance of NASNetLarge in terms of precision and F1-score shows its promise as a reliable aid for the accurate detection and classification of cardiac events in lead aVR. The results strongly support the models’ ability to act as reliable diagnostic instruments, setting the stage for better cardiac treatment and intervention.

### Appendix A.5. Performance Evaluation for Lead aVL

Lead aVL is an important limb lead in the ECG, giving a view of the heart’s electrical activity as seen from the angle of the left arm. It is very important for the detection of lateral myocardial infarction and cardiac abnormalities that involve the left side of the heart. The exact interpretation of this lead is important for correct cardiac diagnosis, particularly for problems affecting the lateral wall of the left ventricle. The assessment of the chosen deep learning models for lead aVL produced the insights shown in Table A5.

**Table A5:** Performance on lead aVL.

Performance	Model Name	Lead I				Mean
		Normal	Abnormal	PMI	MI	
Precision	NASNetLarge	90.91%	96.08%	96.97%	95.22%	94.79%
	Inception	89.54%	92.52%	91.57%	98.76%	93.10%
	MobileNetV2	93.17%	95.35%	93.18%	97.54%	94.81%
	Densnet201	93.27%	96.67%	89.50%	99.58%	94.75%
	VGG16	92.59%	96.74%	94.12%	97.15%	95.15%
Recall	NASNetLarge	98.59%	84.12%	93.02%	100.00%	93.93%
	Inception	96.48%	84.98%	88.37%	100.00%	92.46%
	MobileNetV2	96.13%	87.98%	95.35%	99.58%	94.76%
	Densnet201	97.54%	87.12%	94.19%	100.00%	94.71%
	VGG16	96.83%	89.27%	93.02%	100.00%	94.78%
F1-Score	NASNetLarge	94.59%	89.70%	94.96%	97.55%	94.20%
	Inception	92.88%	88.59%	89.94%	99.38%	92.70%
	MobileNetV2	94.63%	91.52%	94.25%	98.55%	94.74%
	Densnet201	95.35%	91.65%	91.78%	99.79%	94.64%
	VGG16	94.66%	92.86%	93.57%	98.56%	94.91%

To explore the major performance of the deep learning models, we can conclude the results of lead aVL as follows:

- Precision: VGG16 demonstrated outstanding precision, leading with 95.15%, suggesting a superior ability to accurately identify true positives in lead aVL. NASNetLarge and MobileNetV2 followed closely, with precision rates of 94.97% and 94.81%, respectively, indicating their reliable classification performance in detecting specific cardiac conditions from this lead.
- Recall: For recall, VGG16 was the top performer, with a 94.78% mean recall rate, indicating a high sensitivity to actual positive cases. Densnet201 and MobileNetV2 also showed strong recall abilities, with mean rates of 94.71% and 94.76%, respectively, reinforcing their capability to minimize false negatives in diagnoses.
- F1-Score: The F1-score, which balances precision and recall, saw VGG16 achieve the highest mean score of 94.91%. This indicates a harmonious balance between the model’s precision and recall, signifying its suitability for the reliable detection of cardiac anomalies in lead aVL. DenseNet201 and MobileNetV2 also recorded high F1-scores, reflecting their effectiveness in accurate diagnosis.

These results for lead aVL illustrate the models’ potential in clinical settings, with VGG16 standing out for its precision and balanced accuracy. The high-performance metrics across the board for DenseNet201 and MobileNetV2 emphasize the effectiveness of these models for cardiac diagnosis when interpreting data from lead aVL.

### Appendix A.6. Performance Evaluation for Lead aVF

A unipolar lead in the frontal plane of the ECG, called lead aVF, is vital for the detection of inferior heart conditions. Due to its specific anatomical position, it plays a key role in the diagnosis of conditions including inferior myocardial infarctions and other cardiac irregularities. A detailed analysis of this lead’s data is important because of its clinical relevance. The experiment assessed the performance of a range of deep learning models in the interpretation of ECG data from lead aVF. A careful analysis yielded an evaluation of model performance on lead aVF, which is organized in Table A6.

**Table A6:** Performance on lead aVF.

Performance	Model Name	Lead I				Mean
		Normal	Abnormal	PMI	MI	
Precision	NASNetLarge	89.03%	93.90%	89.38%	97.55%	92.46%
	Inception	93.71%	92.27%	85.64%	99.17%	92.70%
	MobileNetV2	92.20%	94.71%	83.52%	98.35%	92.20%
	Densnet201	92.88%	94.59%	91.02%	97.95%	94.11%
	VGG16	93.65%	95.07%	93.33%	99.17%	95.30%
Recall	NASNetLarge	97.18%	85.84%	83.14%	100.00%	91.54%
	Inception	94.37%	87.12%	90.12%	100.00%	92.90%
	MobileNetV2	95.77%	84.55%	88.37%	100.00%	92.17%
	Densnet201	96.48%	90.13%	88.37%	100.00%	93.74%
	VGG16	98.59%	90.99%	89.53%	100.00%	94.78%
F1-Score	NASNetLarge	92.93%	89.69%	86.14%	98.76%	91.88%
	Inception	94.04%	89.62%	87.82%	99.58%	92.77%
	MobileNetV2	93.96%	89.34%	85.88%	99.17%	92.09%
	Densnet201	94.65%	92.31%	89.68%	98.96%	93.90%
	VGG16	96.05%	92.98%	91.39%	99.58%	95.00%

To explore the major performance of the deep learning models, we can conclude the results of lead aVF as follows:

- Precision: Our models demonstrated commendable precision across all conditions, with VGG16 leading at a mean precision of 95.30%. This high precision showcases VGG16’s capability to accurately identify true positive cases. DenseNet201 and Inception were not far behind, both demonstrating significant precision, as per the table results.
- Recall: VGG16 emerged as the superior model in recall as well, with a mean recall of 94.78%. This model’s proficiency lies in its ability to capture the majority of actual cases, minimizing false negatives. DenseNet201 closely followed, indicating its heightened sensitivity across the board for conditions depicted by lead aVF.
- F1-Score: The F1-score, which harmonizes precision and recall, indicates the models’ balanced accuracy. VGG16 obtained the highest mean F1-score, at 95.00%, closely trailed by DenseNet201, at 93.90%. These scores from the table point to the models’ competence in reliably detecting cardiac anomalies using lead aVF data.

The aggregated data for lead aVF show admirable performance across all models, with VGG16 and DenseNet201 displaying exceptional efficacy. VGG16’s robust precision and recall make it a reliable model, especially in critical diagnostic settings. DenseNet201’s balanced F1-score underlines its utility in accurately diagnosing both the presence and absence of cardiac conditions, making it highly suitable for clinical assessments.

### Appendix A.7. Performance Evaluation for Lead V1

Lead V1 is located in the fourth intercostal space to the right of the sternum, delivering important information about the right ventricular and septal parts of the heart. It is notably useful in diagnosing conditions affecting the right side of the heart, anterior septal infarctions, and arrhythmias. Correct analysis of lead V1 data is important for the early recognition and adequate management of these cardiac problems. The assessment of the chosen deep learning models on lead V1 included detailed computation of precision, recall, and F1-scores for all conditions. The provided Table A7 summarizes the outcomes as follows.

**Table A7:** Performance on lead V1.

Performance	Model Name	Lead I				Mean
		Normal	Abnormal	PMI	MI	
Precision	NASNetLarge	91.95%	96.63%	88.70%	97.55%	93.71%
	Inception	93.45%	94.91%	90.80%	96.37%	93.88%
	MobileNetV2	95.37%	92.11%	91.43%	97.95%	94.21%
	Densnet201	92.52%	95.24%	87.15%	97.55%	93.11%
	VGG16	93.56%	96.28%	90.23%	97.95%	94.50%
Recall	NASNetLarge	96.48%	86.27%	91.28%	100.00%	93.51%
	Inception	95.42%	87.98%	91.86%	100.00%	93.82%
	MobileNetV2	94.37%	90.13%	93.02%	100.00%	94.38%
	Densnet201	95.77%	85.84%	90.70%	100.00%	93.08%
	VGG16	97.18%	88.84%	91.28%	100.00%	94.33%
F1-Score	NASNetLarge	94.16%	91.16%	89.97%	98.76%	93.51%
	Inception	94.43%	91.31%	91.33%	98.15%	93.81%
	MobileNetV2	94.87%	91.11%	92.22%	98.96%	94.29%
	Densnet201	94.12%	90.29%	88.89%	98.76%	93.02%
	VGG16	95.34%	92.41%	90.75%	98.96%	94.37%

To explore the major performance of the deep learning models, we can conclude the results of lead V1 as follows:

- Precision: VGG16 showed the highest mean precision, at 94.50%, indicating its excellent capability for precise positive case identification in lead V1. MobileNetV2 followed with a mean precision of 94.21%, displaying strong performance in true positive classification.
- Recall: For recall, MobileNetV2 displayed superior performance, with a mean rate of 94.38%, suggesting its strong ability to capture the majority of actual cases, which is crucial for clinical diagnostics. VGG16 also demonstrated high recall ability, with a mean rate of 94.33%.
- F1-Score: When considering the F1-score, VGG16 achieved the highest mean score of 94.37%, indicating a strong balance between precision and recall. MobileNetV2 came close, with a mean F1-score of 94.29%, showing that it maintains balanced accuracy in the detection of cardiac anomalies as presented in lead V1.

The assessment of lead V1 highlights the capability of MobileNetV2 and VGG16 as top performers in all three metrics, underscoring their potential as reliable tools for the diagnosis of cardiac conditions through this specific ECG lead. The comprehensive performance metrics from the table affirm these models’ suitability for incorporation into clinical ECG analysis systems, potentially enhancing diagnostic accuracy for conditions detected through lead V1.

### Appendix A.8. Performance Evaluation for Lead V2

Lead V2 is placed symmetrically to lead V1 on the left side of the sternum, and offers an in-depth look into the septal wall of the heart. This lead is essential for detecting abnormalities in the septal region, which can be indicative of septal infarcts or other types of cardiac events. As such, lead V2 is pivotal for comprehensive cardiac assessment, and accurate model interpretation of its readings is of significant clinical importance. The deep learning models’ ability to classify cardiac conditions using lead V2 was extensively examined, with the following results, summarized in Table A8.

**Table A8:** Performance on lead V2.

Performance	Model Name	Lead I				Mean
		Normal	Abnormal	PMI	MI	
Precision	NASNetLarge	96.60%	97.24%	95.95%	97.95%	96.93%
	Inception	96.25%	98.15%	94.97%	99.58%	97.24%
	MobileNetV2	96.55%	96.02%	97.09%	99.58%	97.31%
	Densnet201	95.90%	96.86%	96.51%	99.58%	97.22%
	VGG16	97.58%	97.32%	95.98%	99.17%	97.51%
Recall	NASNetLarge	100.00%	90.56%	96.51%	100.00%	96.77%
	Inception	99.30%	90.99%	98.84%	100.00%	97.28%
	MobileNetV2	98.59%	93.13%	97.09%	100.00%	97.20%
	Densnet201	98.94%	92.70%	96.51%	100.00%	97.04%
	VGG16	99.30%	93.56%	97.09%	100.00%	97.49%
F1-Score	NASNetLarge	98.27%	93.78%	96.23%	98.96%	96.81%
	Inception	97.75%	94.43%	96.87%	99.79%	97.21%
	MobileNetV2	97.56%	94.55%	97.09%	99.79%	97.25%
	Densnet201	97.40%	94.74%	96.51%	99.79%	97.11%
	VGG16	98.43%	95.40%	96.53%	99.58%	97.49%

To explore the major performance of the deep learning models, we can conclude the results of lead V2 as follows:

- Precision: VGG16 achieved the highest mean precision, at 97.51%, indicating exceptional accuracy in classifying true positive cases from lead V2. MobileNetV2 also demonstrated high precision, at 97.31%, followed closely by Inception and Densnet201, both exceeding 97%. These results suggest a keen ability of the models to correctly identify the presence of cardiac events in this particular lead.
- Recall: For recall, VGG16 displayed superior performance, with a mean rate of 97.49%, suggesting its strong ability to capture the majority of actual cases, which is crucial for clinical diagnostics. Inception also demonstrated high recall ability, with a mean rate of 97.28%.
- F1-Score: The F1-score, a critical measure of a model’s balanced accuracy, saw VGG16 leading once again, with a mean F1-score of 97.49%. MobileNetV2 and Inception were in close pursuit with their F1-scores, demonstrating their balanced precision and recall.

These findings for lead V2 are significant, highlighting the models’ potential for use in clinical practice, where VGG16 has shown a strong propensity for accuracy across all metrics. The deep learning models’ high precision, recall, and F1-scores underscore their capability to reliably interpret ECG data from lead V2, and suggest that they could play a vital role in advancing cardiac diagnostic procedures.

### Appendix A.9. Performance Evaluation for Lead V3

Lead V3 is strategically placed between V2 and V4, to provide detailed insights into the septal and anterior walls of the left ventricle. This lead is particularly vital for identifying anterior myocardial infarctions and other frontally located cardiac events. Given its positioning, lead V3 is crucial for comprehensive ECG analysis and accurate detection of specific cardiac conditions. Upon evaluating the deep learning models for their performance on lead V3, the following results were observed, recorded in Table A9.

**Table A9:** Performance on lead V3.

Performance	Model Name	Lead I				Mean
		Normal	Abnormal	PMI	MI	
Precision	NASNetLarge	95.88%	95.91%	96.00%	98.76%	96.64%
	Inception	94.81%	94.27%	94.77%	99.58%	95.86%
	MobileNetV2	95.50%	94.27%	97.63%	97.53%	96.23%
	Densnet201	94.67%	98.11%	95.95%	98.35%	96.77%
	VGG16	97.57%	97.76%	96.59%	99.17%	97.77%
Recall	NASNetLarge	98.24%	90.56%	97.67%	100.00%	96.62%
	Inception	96.48%	91.85%	94.77%	100.00%	95.77%
	MobileNetV2	97.18%	91.85%	95.93%	99.16%	96.03%
	Densnet201	100.00%	89.27%	96.51%	100.00%	96.45%
	VGG16	98.94%	93.56%	98.84%	100.00%	97.84%
F1-Score	NASNetLarge	97.04%	93.16%	96.83%	99.38%	96.60%
	Inception	95.64%	93.04%	94.77%	99.79%	95.81%
	MobileNetV2	96.34%	93.04%	96.77%	98.34%	96.12%
	Densnet201	97.26%	93.48%	96.23%	99.17%	96.54%
	VGG16	98.25%	95.61%	97.70%	99.58%	97.79%

To explore the major performance of the deep learning models, we can conclude the results of lead V3 as follows:

- Precision: VGG16 stood out with an exemplary mean precision of 97.77%, suggesting its high accuracy in pinpointing true positives within lead V3’s readings. NASNetLarge and DenseNet201 also showed impressive precision, scoring 96.64% and 96.77%, respectively, which highlights their reliability in the accurate classification of cardiac conditions.
- Recall: VGG16 showcased superior performance in recall, with a rate of 97.84%, closely mirroring its precision rate. This indicates VGG16’s effectiveness in capturing most true positive cases—a key feature for a clinical diagnostic tool. NASNetLarge also performed well, with a mean recall rate of 96.62%.
- F1-Score: Considering the F1-score, VGG16 maintained its lead, with a mean score of 97.79%, demonstrating an excellent balance between precision and recall. Both NASNetLarge and DenseNet201 also showcased high F1-scores, at 96.60% and 96.54%, respectively, indicating their effectiveness in providing harmonized accuracy in the detection of cardiac anomalies using lead V3.

The performance assessment of lead V3 accentuates the potential of the analyzed models, especially VGG16, for clinical application. The models’ high precision, recall, and F1-scores establish them as potent tools for reliable interpretation of ECG data, and suggest their viability in aiding and enhancing cardiac diagnostic procedures.

### Appendix A.10. Performance Evaluation for Lead V4

Lead V4 is positioned in the fifth intercostal space at the midclavicular line, directly over the left ventricle. It is key in diagnosing conditions affecting the anterior wall of the heart, and is particularly sensitive to changes in this region. Given its critical placement, the accuracy of data interpretation from lead V4 is paramount for identifying myocardial infarctions and other anterior cardiac anomalies. The evaluation of our deep learning models on lead V4 produced the following results, as explained in Table A10.

**Table A10:** Performance on lead V4.

Performance	Model Name	Lead I				Mean
		Normal	Abnormal	PMI	MI	
Precision	NASNetLarge	95.53%	95.87%	95.43%	97.95%	96.20%
	Inception	96.50%	97.26%	95.51%	97.55%	96.71%
	MobileNetV2	96.54%	96.88%	96.00%	99.58%	97.25%
	Densnet201	96.50%	97.29%	95.56%	99.17%	97.13%
	VGG16	97.92%	98.21%	97.14%	99.17%	98.11%
Recall	NASNetLarge	97.89%	89.70%	97.09%	100.00%	96.17%
	Inception	97.18%	91.42%	98.84%	100.00%	96.86%
	MobileNetV2	98.24%	93.13%	97.67%	100.00%	97.26%
	Densnet201	97.18%	92.27%	100.00%	100.00%	97.36%
	VGG16	99.30%	94.42%	98.84%	100.00%	98.14%
F1-Score	NASNetLarge	96.70%	92.68%	96.25%	98.96%	96.15%
	Inception	96.84%	94.25%	97.14%	98.76%	96.75%
	MobileNetV2	97.38%	94.97%	96.83%	99.79%	97.24%
	Densnet201	96.84%	94.71%	97.73%	99.58%	97.22%
	VGG16	98.60%	96.28%	97.98%	99.58%	98.11%

To explore the major performance of the deep learning models, we can conclude the results of lead V4 as follows:

- Precision: The precision metric was dominated by VGG16, which achieved an extraordinary mean precision of 98.11%. MobileNetV2 also performed excellently, with a mean precision of 97.25%, closely followed by DenseNet201, at 97.13%. These high precision rates reflect the models’ capability to correctly classify true positives, a crucial aspect of cardiac event detection.
- Recall: For recall, VGG16 showed superior performance, with an impressive mean recall of 98.14%. DenseNet201 and MobileNetV2 also exhibited strong recall abilities, with mean scores of 97.36% and 97.26%, respectively. These figures indicate the models’ strong capability to identify most true positive cases, minimizing the likelihood of missed diagnoses.
- F1-Score: VGG16 maintained a leading position in the F1-score metric, with a mean of 98.11%, showcasing an excellent balance between precision and recall. DenseNet201 and MobileNetV2 both demonstrated high F1-scores as well, with mean values of 97.22% and 97.24%, respectively, indicating their effective and balanced classification performance in lead V4.

The lead V4 performance evaluation suggests that VGG16 may be especially suitable for clinical applications, with its high precision and recall indicating its potential as a reliable tool for cardiac diagnosis using this ECG lead. The high performance across the board for models like MobileNetV2 and DenseNet201 emphasizes the effectiveness of these models for cardiac diagnosis when interpreting data from lead V4. These models’ capacity for high precision, recall, and F1-scores highlight their potential to significantly improve the accuracy of ECG-based cardiac diagnostics.

### Appendix A.11. Performance Evaluation for Lead V5

Lead V5, positioned on the horizontal plane at the left anterior axillary line, is crucial for the assessment of the lateral wall of the left ventricle. It is an important lead for identifying lateral myocardial infarctions, and can be indicative of left ventricular hypertrophy and other conditions that may not be visible in other leads. Accuracy and precision in analyzing lead V5 are vital for comprehensive cardiac analysis. The models were evaluated for their performance on lead V5, as presented in Table A11.

**Table A11:** Performance on lead V5.

Performance	Model Name	Lead I				Mean
		Normal	Abnormal	PMI	MI	
Precision	NASNetLarge	93.69%	94.44%	93.57%	99.58%	95.32%
	Inception	94.41%	92.92%	94.19%	97.95%	94.87%
	MobileNetV2	96.53%	96.38%	92.74%	99.58%	96.31%
	Densnet201	94.24%	95.89%	92.44%	98.76%	95.33%
	VGG16	95.86%	96.43%	96.47%	97.95%	96.68%
Recall	NASNetLarge	99.30%	87.55%	93.02%	100.00%	94.97%
	Inception	95.07%	90.13%	94.19%	100.00%	94.85%
	MobileNetV2	97.89%	91.42%	96.51%	100.00%	96.45%
	Densnet201	97.89%	90.13%	92.44%	100.00%	95.11%
	VGG16	97.89%	92.70%	95.35%	100.00%	96.49%
F1-Score	NASNetLarge	96.41%	90.87%	93.29%	99.79%	95.09%
	Inception	94.74%	91.50%	94.19%	98.96%	94.85%
	MobileNetV2	97.20%	93.83%	94.59%	99.79%	96.35%
	Densnet201	96.03%	92.92%	92.44%	99.38%	95.19%
	VGG16	96.86%	94.53%	95.91%	98.96%	96.57%

To explore the major performance of the deep learning models, we can conclude the results of lead V5 as follows:

- Precision: VGG16 showed exceptional precision, with a mean value of 96.68%, reflecting its ability to accurately identify true positive cases. MobileNetV2 also demonstrated high precision, coming in at 96.31%, indicative of its effective and accurate classification capabilities within lead V5’s context.
- Recall: In terms of recall, VGG16 achieved a remarkable score of 96.49%, displaying its strength in recognizing the majority of actual positive cases. MobileNetV2 followed closely, with a recall rate of 96.45%, underscoring its proficiency in detecting true cases and minimizing the risk of false negatives.
- F1-Score: Regarding the F1-score, which provides a balance between precision and recall, VGG16 achieved a mean of 96.57%, highlighting its efficient diagnostic performance. MobileNetV2 also presented a high F1-score of 96.35%, confirming its status as a well-balanced and accurate model for analyzing lead V5 data.

The evaluation of lead V5 points to VGG16 and MobileNetV2 as potential frontrunners for clinical applications, with their high scores in precision, recall, and F1-score underscoring their capabilities to serve as reliable tools for cardiac diagnosis. The results exhibit the strengths of the deep learning models in providing accurate interpretations of ECG data, suggesting their possible integration into clinical diagnostic workflows for enhanced cardiac event detection and monitoring.

### Appendix A.12. Performance Evaluation for Lead V6

Lead V6, positioned at the midaxillary line at the same horizontal level as V4 and V5, offers a crucial perspective on the lateral wall of the left ventricle. Its interpretation is essential for diagnosing lateral myocardial infarctions and for providing a comprehensive view of the heart’s electrical activity. Precision in lead V6 analysis is key for a full understanding of the heart’s condition, particularly for lateral cardiac events. In our continued evaluation of deep learning models, lead V6 presented the following key performance results, as shown in Table A12.

**Table A12:** Performance on lead V6.

Performance	Model Name	Lead I				Mean
		Normal	Abnormal	PMI	MI	
Precision	NASNetLarge	94.43%	92.86%	92.44%	96.73%	94.11%
	Inception	91.33%	93.69%	94.44%	97.95%	94.36%
	MobileNetV2	93.52%	94.06%	92.61%	99.58%	94.94%
	Densnet201	94.81%	96.41%	93.64%	98.35%	95.80%
	VGG16	95.21%	95.52%	94.74%	98.76%	96.05%
Recall	NASNetLarge	95.42%	89.27%	92.44%	99.16%	94.07%
	Inception	96.48%	89.27%	88.95%	100.00%	93.68%
	MobileNetV2	96.48%	88.41%	94.77%	100.00%	94.91%
	Densnet201	96.48%	92.27%	94.19%	100.00%	95.73%
	VGG16	97.89%	91.42%	94.19%	100.00%	95.87%
F1-Score	NASNetLarge	94.92%	91.03%	92.44%	97.93%	94.08%
	Inception	93.84%	91.43%	91.62%	98.96%	93.96%
	MobileNetV2	94.97%	91.15%	93.68%	99.79%	94.90%
	Densnet201	95.64%	94.30%	93.91%	99.17%	95.75%
	VGG16	96.53%	93.42%	94.46%	99.38%	95.95%

To explore the major performance of the deep learning models, we can conclude the results of lead V6 as follows:

- Precision: VGG16 emerged with the highest mean precision of 96.05%, indicating its significant accuracy in correctly identifying true positives from lead V6. DenseNet201 also showcased high precision, with a mean of 95.80%, while MobileNetV2 held a mean precision of 94.94%, both indicating strong classification performance.
- Recall: VGG16 led the recall metric, with a mean rate of 95.87%, reflecting its exceptional ability to capture the majority of actual cases, a crucial capability in clinical diagnostics to avoid missed diagnoses. DenseNet201 followed closely, with a mean recall of 95.73%, confirming its effectiveness in detecting true positive cases.
- F1-Score: Regarding the F1-score, VGG16 maintained the lead, with a mean of 95.95%, signifying a superior balance between precision and recall. DenseNet201, with a mean F1-score of 95.75% and MobileNetV2, at 94.90% also displayed commendable balanced accuracy, which is important for a dependable diagnostic process.

The assessment of performance for lead V6 shows that VGG16 has the potential to be a stable tool for clinical applications, scoring high in precision, recall, and F1-score. The findings for DenseNet201 and MobileNetV2 show that they are capable models, with great promise in helping with the correct interpretation of ECG data from lead V6. These results bring attention to developments in cardiac diagnostic technology and the bright future of machine learning in cardiology.

Our comprehensive assessment of performance includes our suite of deep learning models—NASNetLarge, Inception, DenseNet201, MobileNetV2, and VGG16—across all individual ECG leads—V1 to V6, aVF, aVL, and aVR. The accuracy, recall, and F1-score measurements from these models yield significant understanding of their diagnostic capabilities. VGG16 regularly showed up as a leading performer, often leading in precision, recall, and F1-scores across the majority of leads. Its superior performance points to its potential as a strong aid for the precise interpretation of ECG data and for dependable cardiac diagnosis in healthcare environments.

DenseNet201 and MobileNetV2 demonstrated impressive capabilities, regularly performing nearly as well as, or occasionally surpassing, VGG16 in a variety of metrics. Their performances suggest that they could play a key role in clinical analysis of ECG leads, particularly in cases where a range of models may facilitate comprehensive diagnostics. The detailed investigation of each lead uncovered special understanding of the models’ capabilities. In leads where precision is important to eliminate false positives, including V2 and V5, the models provided very reliable case identification. In situations where recall is important to reduce false negatives, as in V3 and V6, the models showed impressive sensitivity.

The F1-scores showed that the models kept a harmonious balance between precision and recall, an essential requirement in clinical diagnostics to make sure that both the presence and absence of conditions are correctly diagnosed. The reliably high mean scores across all leads show the promise of these models to fit into cardiac diagnostic workflows, supporting the skill of clinicians. The results from Experiment 1 demonstrate the progress made in applying deep learning to cardiology, suggesting that these models may be able to help in the early detection, intervention, and treatment of cardiac conditions. The findings indicate that as we move forward with AI in healthcare, it will likely have a transformative effect on cardiac care, helping to improve results and save lives.

## Appendix B. Supplementary Training Curves

Figure A1a–e and Figure A2a–e illustrate the training and validation accuracy/loss curves for VGG16 and MobileNetV2 with lead V4 across five-fold cross-validation.

### Appendix B.2. Accuracy/Loss Curves for MobileNetV2 with Lead V4

The inclusion of these curves provides additional insight into the models’ training behaviors, showcasing their learning dynamics and generalizability.
